# Wildlife Diseases: Pathology and Diagnostic Investigation

**DOI:** 10.3390/ani16091343

**Published:** 2026-04-28

**Authors:** Lorenzo Domenis, Serena Robetto

**Affiliations:** Istituto Zooprofilattico Sperimentale del Piemonte, Liguria e Valle d’Aosta, Via Bologna 148, 10154 Torino, Italy; serena.robetto@izsplv.it

In recent years, especially following the COVID-19 outbreak, wildlife studies and publications have focused on the roles of free-living animal species in the spillover of pathogens towards humans, livestock, and pets [1]. Recognizing the importance of this epidemiological aspect—widely acknowledged by the scientific community within One Health [2]—this Special Issue aimed to reconsider wild fauna as a genuine subject of veterinary and medico-legal investigation beyond its role as the (often healthy) carrier of zoonotic agents. From this perspective, we favored and selected articles reporting original cases of pathological processes (congenital, metabolic–degenerative, parasitic, infectious, and neoplastic) in free-ranging or captive wild animals (including exotic species), without neglecting non-pathological conditions (e.g., trauma and starvation), as well as studies related to their anatomical and physiological aspects. Special attention was devoted to the description of necropsy and histopathological findings, particularly important tools for supporting the damaging action of etiological factors identified and confirmed through strong diagnostic protocol (including bacteriological and virological examinations, biomolecular techniques, and clinical chemistry) and, ultimately, for distinguishing infectious diseases from asymptomatic infections.

Having evaluated the 24 manuscripts on the basis of animal species, marine mammals appear to have mainly been studied, including six articles referring to dugong (*Dugong dugon*), harbor seal (*Phoca vitulina*), narrow-ridged finless porpoise (*Neophocaena asiaeorientalis sunameri*) and different species of dolphins, whales, and wild ruminants, with six articles referring to oryx (*Oryx leucoryx*), different species of cervids, ibex (*Capra ibex*), and white-tailed deer (*Odocoileus virginianus*). The greater attention given to detecting diseases or abnormalities in these animal categories may be explained by considering the fact that they are frequently the objects of conservation projects with targeted surveillance (such as oryx and cetaceans), or, more generally, that large animal carcasses are more easily found in nature by humans (tourists, forest rangers, scholars, or naturalists), such as in the case of marine mammal strandings. When grouping the articles based on pathological process, reports about infectious and parasitic diseases prevail, with ten articles referring to *Achromobacter xylosidans* in dugong, the foot-and-mouth disease virus in oryx, *Brucella ceti* in cetaceans, orthomyxovirus H5N1 in Antarctic birds, *Mycobacterium* spp. in badger (*Meles meles*) and white-tailed deer, *Escherichia coli/Mycoplasma conjunctivae* co-infection in ibex, *Aspergillus fumigatus* in scarlet macaw parrot (*Ara Macau*), different bacteria in the lung of nutria (*Myocastor coypus*), and parasitic infections in whales and dolphins. These are followed by the topic of neoplastic diseases, with six articles referring to cholangiocarcinoma in puma (*Puma concolor*), gallbladder carcinoma in otter (*Lutra lutra*), squamous cell carcinoma in African pygmy hedgehog (*Atelerix albiventris*), epidermoid cyst in porcupine (*Hystrix cristata*), lymphoma in red squirrel (*Sciurus vulgaris*) and different tumor types and tumor-like lesions in fox (*Vulpes vulpes*). If the infection—often asymptomatic (as it is for mycobacteria and orthomyxovirus H5N1)—by parasites, bacteria and viruses matches what we could logically hypothesize in free-ranging animals exposed to a hostile environment without veterinary care, the sequence of neoplasms in wildlife species deserves particular attention. Globally, wild animals share the same carcinogenic factors active in humans due to reduced natural habitats and increasing pollution, and can therefore actually be involved in neoplastic processes [3]. The growing interest in comparative oncology, traditionally oriented towards domestic animals (mainly dogs and cats) where spontaneous tumors are more frequent, is stimulating attention to alternative animal models among wild fauna. Apart from the known endemic neoplasms like, e.g., the facial tumor in Tasmanian devils (*Sarcophilus harrisii*), fibropapillomatosis in sea turtles (*Chelonia mydas*) and urogenital carcinoma in California sea lions (*Zalophus californianus*) [4], neoplastic diseases in wild animals are generally rather rare and obviously difficult to investigate due to the lack of anamnesis and evident impossibility of therapeutic/follow up verification. However, this very aspect makes them worthy of study, for example regarding possible different mechanisms for suppressing cancer—as found in elephants and sea sponges [5,6]—or the impact of carcinogenic factors in apparently pollution-free natural habitats, as observed in belugas (*Delphinapterus leucas*). Serving as evidence of the growing interest that the scientific community is dedicating to wild animals even on less conventional topics, the Special Issue is completed by some articles that deal with aspects not linked to traditional infectious or neoplastic diseases, such as congenital anomalies of cervid horns, dental and skull-bone pathologies of the racoon dog (*Nyctereutes procyonoides*); organic lesions due to sand impaction in white-tailed deer and adhesive intestinal obstruction in narrow-crested finless porpoise; particular anatomical or physiological features, such as the reference values for post-mortem examinations of the hearts of macropods (*Macropodidae*) and koalas (*Phascolarctidae*); and intracytoplasmic hepatocytes eosinophilic globules in cetaceans. Apart from cases of specific diseases or abnormalities, the main causes of death—especially in medium and small wild animals—remain traumatic events related to human activities (e.g., impact with vehicles and getting caught in fishing nets)—as evidenced not only by the present survey on the European hedgehog (*Erinaceus europaeus*), but also by other studies on, for example, otters, wolves and turtles—and starvation due a fatal combination of different and overlapping pathological processes (viral and bacteriological infections, parasitic diseases, pollution), especially when associated with periods of food scarcity [7].

As broadly evidenced by most articles in the Special Issue, the diagnostic activity on wild animals involves the application of hard protocol, where multiple disciplines, from biology and natural science to pathological anatomy and the most advanced biotechnological techniques, must be integrated to achieve the final goal; in other words, establishing the cause of death [8]. Diagnostic work, especially that performed on wild animals found dead in the environment, is rather complex and difficult; in the absence of any kind of anamnesis (as in the case of zoo animals or captive wildlife), the investigation is based exclusively on the anatomical–pathological findings and subsequent laboratory exams. Analyses, particularly bacteriological and histological exams, are often compromised by the poor quality of tissue, considering the fact that the time between death and finding the carcass is often particularly long, and even more so if the weather conditions are adverse (as can happen in summer for high temperatures). The lack of validated tests for wildlife, especially with regard to serological diagnostics, contributes to this difficulty [9]. Therefore, all case reports where advantageous circumstances (favorable climate, the absence of post-mortem animal predation, timely collection and analysis of the carcass) allowed for the achievement of a reliable diagnostic result are to be considered valuable opportunities that truly improve the knowledge of wildlife diseases.

In conclusion, we hope this Special Issue will be a valuable tool for training and educating future professionals involved in the study and management of wildlife—above all naturalists, foresters, veterinarians, and forensic pathologists—with a special perspective towards three main objectives; in other words, biodiversity and ecosystem conservation (the prevention of diseases as well as criminal activity, e.g., poaching, equally contributes to the welfare of wild populations and in particular of endangered species), the health of livestock and pets (threatened by the interspecies spillover of pathogens shared between domestic and wild fauna), and last but not least, public health protection (monitoring the health status of wildlife avoids the transmission of infectious agents by free-ranging animals to humans).

## Data Availability

No new data were created or analyzed in this study. Data sharing is not applicable to this article.
